# Dr. H. S. Swamy

**Published:** 2011

**Authors:** P. Satishchandra, A. B. Taly

**Affiliations:** Department of Neurology, National Institute of Mental Health & Neurosciences, Bangalore, India

**Figure:**
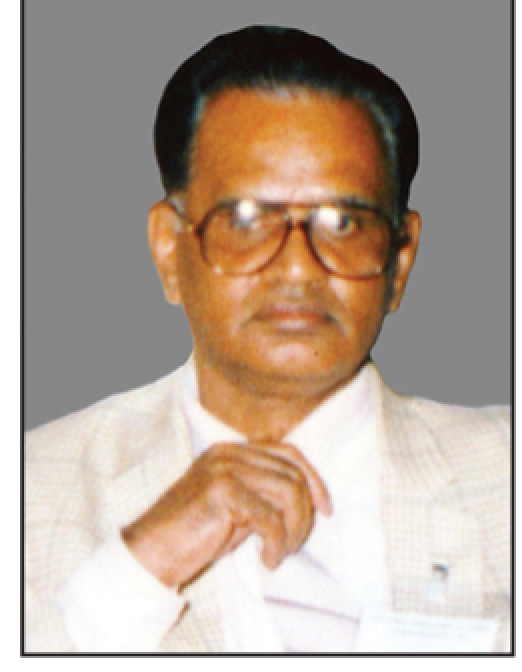
Dr. H. S. Swamy (1938–2010)

It is with profound grief that we bring you the news of the sad demise of Dr. H. Satyanarayan Swamy, former Professor and Head of the Department of Neurology at National Institute of Mental Health & Neurosciences (NIMHANS), Bangalore.

Popularly known to most of us as “HSS”, he will always be remembered by his students as a caring and compassionate physician and a committed teacher who was instrumental in training more than 100 neurologists of our country. His unwavering dedication to serve the needy patients will continue to inspire many of us.

Born in Hassan District of Karnataka on 9 May 1938, he interned in General Medicine and joined the Karnataka State Government services in 1968. His career in neurology began in NIMHANS. He had the distinction of being the very first postgraduate to enroll in the DM Neurology course at NIMHANS. Soon after completion of his training in neurology, he was absorbed as a faculty at NIMHANS in the year 1974 and continued to serve the institute till his superannuation in May 2001.

A dedicated teacher, he will be remembered by his students for his systematic approach and bedside clinics. Adept at all aspects of neurology, most remarkable was his knowledge of neuroradiology, infections of nervous system and movement disorders. He used to emphasize on treatable neurological disorders and the need for building human resource for reaching the community. He used to teach neurology with equal zeal and patience to the postgraduates of internal medicine, psychiatry and pediatrics from other Institutions visiting NIMHANS. His enthusiasm to continue to learn the subject was reflected in his regular presence and active participation in scientific meetings, both at regional and national level, with almost religious fervor.

A compassionate physician, he never discriminated, but cared for both the poor and the not so poor alike. Dr. Swamy had untiring stamina and unwavering dedication in providing services to patients. His genuine concern for patients is reflected in the great lengths he would go to ensure that every needy patient received due attention and free medications from the institute.

Despite his demanding schedule for patient care, he also strongly believed in fostering interest in clinical research in young postgraduates. His belief that clinical research should essentially be a byproduct of patient care services is evident in the nearly 40 publications in national and international journals. His noteworthy contributions in the field are related to Wilson’s disease and anti-rabies vaccine related neurological complications, periodic paralysis and neurological manifestations of *Salmonella* infection.

Superannuating after 33 years of committed service to the state, Dr. Swamy had been serving as Professor of Neurology at KIMS Hospital, Bangalore until a few months back. Following a brief medical illness, Dr. H. S. Swamy, passed away on 17 September 2010 at his residence in Bangalore. He is survived by wife and three children. The neurology fraternity has lost in Dr. Swamy, a teacher and a dedicated physician who truly epitomized compassion.

